# Integrating Physical Activity Strategies to Lower Hyperglycaemia in Structured Education Programmes for Children and Young People with Type 1 Diabetes Improves Glycaemic Control without Augmenting the Risk of Hypoglycaemia

**DOI:** 10.1155/2023/2519368

**Published:** 2023-07-05

**Authors:** John Stuart Pemberton, Ankita Gupta, Gar Mun Lau, India Dickinson, Pranav Viswanath Iyer, Suma Uday

**Affiliations:** ^1^Department of Endocrinology and Diabetes, Birmingham Women's and Children's NHS Foundation Trust, Birmingham, UK; ^2^University of Birmingham, Birmingham, UK; ^3^Institute of Metabolism and Systems Research, College of Medical and Dental Sciences, University of Birmingham, Birmingham, UK

## Abstract

**Objectives:**

Investigate the effect of using short bursts of moderate-intensity activity between meals to lower hyperglycaemia on glucose metrics. *Design and Methods*. Children and young people with type 1 diabetes (CYPD) attending continuous glucose monitoring education were taught to use moderate-intensity activity to lower high glucose levels (to <10.0 mmol/L using 10–15 minlowers ∼2.0 mmol/L) between meals. Retrospective cross-sectional data analysis of CYPD at a single tertiary centre between 2019 and 2022. Data were collected on demographics and glucose metrics (HbA1c, time in range (TIR, 3.9–10.0 mmol/L), time above range (TAR, >10.0 mmol/L), time below range (TBR, <3.9 mmol/L)). Minutes of activity usually performed to lower a glucose level of 14.0 mmol/L trending steady at 6 months grouped the CYPD into low (<5 min), mild (5–10 min), or moderate (11–20 min) activity groups.

**Results:**

125 (*n* = 53, 40% male) CYPD with a mean (standard deviations) age of 12.3 (±3.7) years and diabetes duration of 7.0 ± 3.7 years were included. HbA1c improved from 58.5 (±8.6) mmol/mol at baseline to 54.9 (±7.2) mmol/mol at 6 months (*p* < 0.001). Low, mild, and moderate activity was reported by 30% (*n* = 37), 34% (*n* = 43), and 36% (*n* = 45), respectively. At 6 months, HbA1c (52.0 vs. 54.3 vs. 59.4 mmol/mol, *p* < 0.001), TIR (68.0% vs. 59.71 vs. 51.1%, *p* < 0.001) and TAR (29.9% vs. 38.3% vs. 45.3%, *p* < 0.001) were significantly different across the moderate, mild, and low activity groups, respectively. No association was found for TBR (2.16% vs. 2.32% vs. 2.58%, *p* = 0.408) across groups.

**Conclusion:**

Increasing the use of moderate-intensity activity to lower hyperglycaemia between meals is associated with improved glucose control without increasing hypoglycaemia for CYPD.

## 1. Introduction

Children and young people with type 1 diabetes (CYPD) partaking in physical activity can experience hypoglycaemia (<3.9 mmol/L) during activity [1] and overnight after activity [2]. Unsurprisingly, fear of hypoglycaemia (FoH) for CYPD can pose a major barrier to being active [3]. Additionally, FoH heightens if the CYPD has experienced nocturnal hypoglycaemia related to physical activity [4]. International consensus guidance for exercise management for CYPD provides recommendations for hypoglycaemia prevention while promoting the long-term benefits of being physically active throughout life [5]. Teaching both exercise-induced hypoglycaemia prevention and the health benefits of exercise are an integral part of formal structured education programmes [6, 7] as well as flexible diabetes self-management education programmes [8]. While education on the prevention of hypoglycaemia is crucial, in our clinical experience, the focus on activity-induced hypoglycaemia may serve to exacerbate the FoH and reduce motivation to partake in activity for some individuals. Additionally, exercise education focusing only on the long-term health benefits may miss reinforcement of short-term rewards [9, 10], such as improvements in time in range (TIR, 3.9–10.0 mmol/L). Additionally, positive reinforcement by parents and health care professionals foster a feeling of control over diabetes for the CYPD.

A different perspective on the value of physical activity emerges upon consideration of the benefits of activities such as walking after a meal. Manohar et al. [11] demonstrated in adults with and without type 1 diabetes that walking (at usual cadence for ∼30 min) soon after eating reduced glucose exposure above 7.8 mmol/L by 113% and 145% in the 4 hr after eating, respectively. The improvement in post-meal glycaemia was achieved without hypoglycaemia for the participants with type 1 diabetes [11]. Regulation of post-meal glycaemia through physical activity enables the adaptation of this concept to lower high glucose levels (time above range, TAR, >10.0 mmol/L). A secondary analysis of individual participant data from four studies (*n* = 120 CYPD) by Riddell et al. [1] showed a mean glucose drop of 4.2 mmol/L after 45–60 min of moderate-intensity cycling or walking. However, given that lack of any planned interventions to prevent hypoglycaemia in these studies, nearly 44% of the CYPD experienced hypoglycaemia [1]. On further stratification based on baseline blood glucose, those starting above 10.6 mmol/L experienced a median drop of 6.1 mmol/L, with 17% (7/41) experiencing hypoglycaemia and for those starting above 11.1 mmol/L, 9% (3/34) experienced hypoglycaemia [1]. Furthermore, one of the primary studies included in the secondary analysis measured glucose at regular intervals and reported an incremental drop in glucose every 15 min of moderate-intensity activity, with 6% of participants experiencing hypoglycaemia after 60 min when starting cycling above 10.0 mmol/L [12]. Hence, it could be deduced that with blood glucose above 10.0 mmol/L between meals, performing 15–45 min of moderate-intensity aerobic activity can lower blood glucose to the target range quickly with a low risk of hypoglycaemia, especially if adopted in short blocks of 15 min as necessitated by baseline hyperglycaemia.

The International Society of Pediatric and Adolescent Diabetes (ISPAD) Exercise Guideline 2022 recommends 15–45 min of moderate-intensity aerobic activity to reduce glucose levels above 10.6 mmol/L between meals to improve TIR with a low risk of hypoglycaemia [5]. This is the first-time exercise guidance has specified using activity as a tool to improve TIR [5].

At our tertiary centre, as part of our continuous glucose monitoring (CGM) structured education programme, the “CGM Academy” initiated in 2019, we have taught the use of 10–15 min moderate activity between meals to lower blood glucose by ∼2.0 mmol/L [13, 14]. We have previously reported the effectiveness of our structured education programme (*n* = 100) and demonstrated that using short bursts of activity to lower hyperglycaemia between meals is one of the strongest predictors of improved TIR [14]. In the current report, we aimed to further evaluate the specific relationship between the level of activity used and its effect on lowering hyperglycaemia and the risk of hypoglycaemia in the CGM academy cohort.

## 2. Aim

To investigate the followings:

1. The effectiveness of short bursts of moderate-intensity activity between meals in lowering hyperglycaemia.
2. The risk of hypoglycaemia with using short bursts of moderate-intensity activity between meals.

## 3. Methods

### 3.1. Study Design

A retrospective analysis of cross-sectional data were collected from CYPD attending the “CGM Academy” at our single tertiary centre.

### 3.2. Study Population

CYPD attending the “CGM Academy” from April 2019 to January 2022 at our centre [14]. All CYPD at Birmingham Children's Hospital who were initiated on CGM as per the National Institute for Health and Care Excellence (NICE) [15] criteria attended the CGM academy. The details of the NICE criteria, as well as the setup of the CGM Academy, have been detailed in our previous reports [13, 14]. In brief, NICE hypoglycaemia criteria for initiation of CGM included (1–5).

1. Severe hypoglycaemia: one episode in the last 6 months requiring glucagon.
2. Asymptomatic hypoglycaemia: more than two episodes of hypoglycaemia (<4.0 mmol/L) per week without symptoms.
3. Impaired hypoglycaemia awareness: more than two episodes of hypoglycaemia (<3.0 mmol/L) per week without symptoms.
4. Nocturnal hypoglycaemia: more than two episodes of hypoglycaemia (<4.0 mmol/L) in the night per week.
5. Fear of hypoglycaemia: actively seeing a psychologist for fear of hypoglycaemia.

Inclusion criteria:

1. CYPD completing the CGM Academy with 6 month data with at least 70% CGM data capture over the preceding 3 months.

Exclusion criteria:

1. CYPD with less than 2 years of diabetes duration due to the honeymoon effect confounding CGM results.
2. CYPD under the age of 5 years due to this cohort not using the planned strategy of using activity to drop glucose levels.

### 3.3. CGM Academy Education Teaching Activity to Lower the Glucose Level

**Figure 1:**
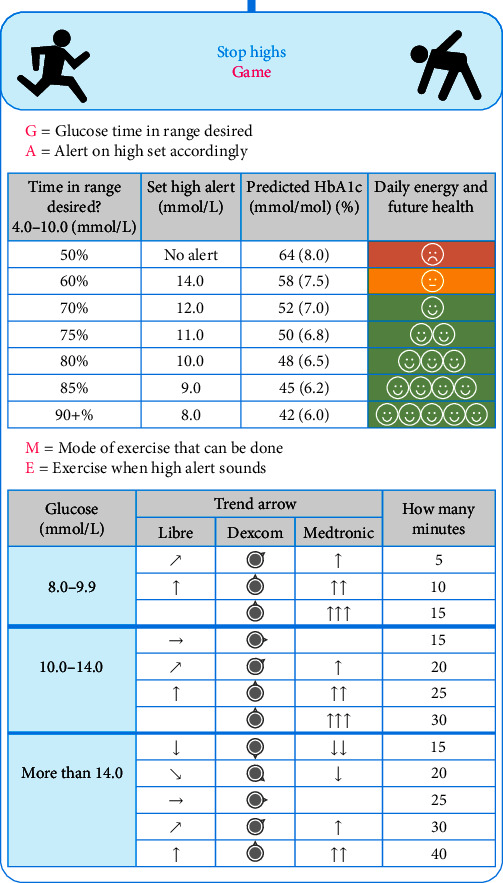
GAME infographic teaching moderate-intensity activity to lower the glucose level between meals.

The CGM Academy delivered by the diabetes team at our centre teaches a range of dynamic glucose management (DynamicGM) strategies over several sessions, which have been previously reported in detail [13, 14]. Exemplars of the teaching are (a) individualised exercise management strategies; (b) hypoglycaemia prevention algorithm based on weight, glucose value, and trend arrow; (c) protocol for pre-meal bolus timing based on glucose value and trend arrow; (d) using moderate-intensity activity to lower high glucose levels between meals [13, 14]. We have detailed the effectiveness of the programme delivered face-to-face and virtually in a previous report [13, 14]. The strategy of using 10–15 min moderate-intensity activity to lower glucose by ∼2.0 mmol/L, aiming to just below 10.0 mmol/L between meals, is briefly summarised using the GAME mnemonic in an infographic (Figure 1); Glucose TIR desired, alert on high set accordingly, mode of moderate-intensity activity (preferred activities), exercise on high alert between meals, if possible, for 5–40 min depending on glucose value and trend arrow. The CYPD and their families were instructed to use preferred activities with an intensity to elicit faster breathing while still being able to speak, such as brisk walking, biking, dancing, playing energetic video games, and playing sports in the garden. All participants started with a high-glucose alert set at 14.0 mmol/L for the structured education programme.

### 3.4. Data Collection

Demographic data, including age, gender, ethnicity, HbA1c (mmol/mol), duration of diabetes, and postcode to determine the quintile of socio-economic deprivation [16], were collected at baseline (start of education) from TWINKLE (online diabetes management database). HbA1c at baseline and 6 months post-education were collected. Data over a 3 month period (starting 3 months after education) on TIR, TAR, TAR2 (>13.9 mmol/L), time below range (TBR), and TBR2 (<3.0 mmol/L) were collected from Dexcom Clarity and Libre View (downloaded at 6-month clinical review).

The use of moderate-intensity activity to lower a glucose level of 14.0 mmol/L that is trending steady to below 10.0 mmol/L between meals was assessed via a patient questionnaire at 6 months [13, 14]. The responses were categorised into three groups based on the time of activity: low activity group (less than 5 min), mild activity group (5–10 min), or moderate activity group (11–20 min).

### 3.5. Data Analysis

All analyses were carried out using SPSS v29.0, with statistical significance set at *p* < 0.05 (two-sided). Descriptive statistics were used to report baseline characteristics (means and standard deviations (SD)) for continuous variables and frequencies and percentages for categorical variables. Dexcom data were expressed as frequencies and percentages. Change in HbA1c from baseline to 6 months was assessed using a paired *t*-test. The 6 month glucose metrics for CYPD using pump therapy was compared to 6 month data for MDI using independent sample *t*-tests. Kruskall–Wallis test was used to compare groups and investigate differences between mean ranks of activity between groups for the outcome variables (HbA1c, TBR2, TBR, TIR, TAR, TAR2). A Mann–Whitney *U* test was used for further group-wise comparison.

## 4. Results

The inclusion criteria were met by 125 (*n* = 53, 42% male) CYPD (excluded: 34 CYPD were within 2 years of diagnosis, seven were <5 years of age, and three had <70% CGM data capture). The mean (SD) age of the cohort was 12.3 (±3.7) years, and diabetes duration of 7.0 ± 3.7 years. The mean baseline HbA1c was 58.5 ± 8.7 mmol/mol. Participants were predominantly of White ethnic background (45%); others included individuals of Asian (31%), Black (12%), and any other ethnic group (12%). The majority of CYPD were using insulin pump therapy (60%), with 40% using multiple daily injections. None of the CYPD were on insulin pumps with predictive low glucose suspend or hybrid closed loop (HCL) functionality. Use of low, mild, and moderate-intensity activity was reported by 30% (*n* = 37), 34% (*n* = 43), and 36% (*n* = 45), respectively.  Table 1 reports the baseline demographic data by activity groups (low, mild, and moderate). There was a significant difference across the groups for age (*p* < 0.01), gender (*p* < 0.05), and socio-economic status (*p* < 0.001). The CYPD in the low group were younger, predominantly females, and were from the most deprived socio-economic quintile when compared to the mild and moderate activity groups.

In the whole cohort, HbA1c improved from 58.5 (±8.6) mmol/mol at baseline to 54.9 (±7.2) mmol/mol at 6 months (*p* < 0.001). At 6 months, HbA1c for CYPD using insulin pump therapy (*n* = 74) was not significantly different to the CYPD using MDI therapy (*n* = 51) (55.4 (±7.4) vs. 54.0 (±6.9) mmol/mol, *p* = 0.30). Similarly, TIR and TAR showed no difference for CYPD using pump therapy and MDI at 6 months (TIR; 60.56 (±12.0)% vs. 59.2 (±12.3)%, *p* = 0.53, TAR; 12.0 (±1.4)% vs. 13.0 (±1.8)%, *p* = 0.53)). Additionally, TBR showed no difference at 6 months (1.8 (±0.2)% vs. 1.6 (±1.8)%, *p* = 0.15). Furthermore, the between-groups comparison in Table 1 shows no difference in the distribution of CYPD using insulin pump therapy and MDI across the three activity groups (*p* = 0.21).

**Figure 2:**
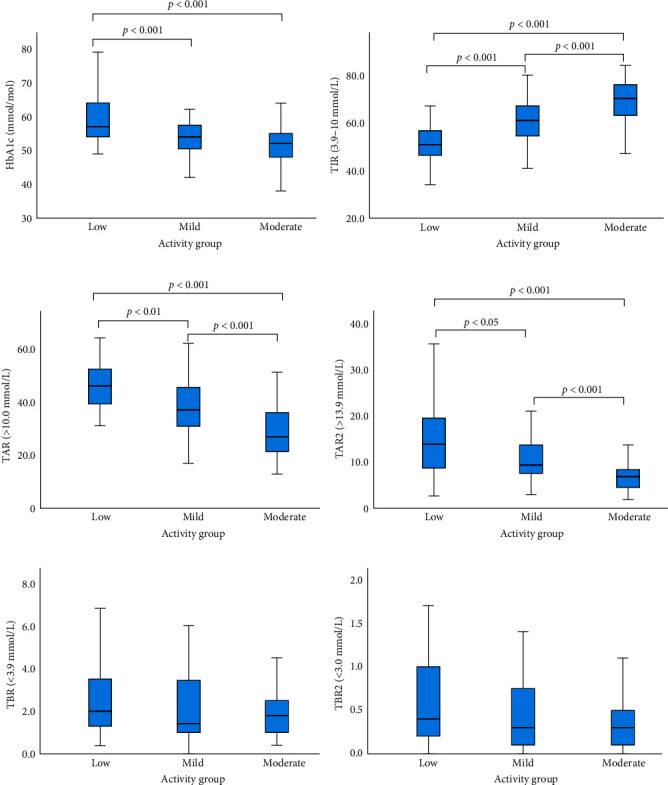
Pairwise comparisons of activity groups (low, mild, and moderate). All significant differences between groups are indicated with *p* values: (a) HbA1c (mmol/mol), (b) time in range (TIR, 3.9–10.0 mmol/L), (c) time above range (TAR, >10.0 mmol/L), (d) time above range 2 (TAR2, >13.9 mmol/L), (e) time below range (TBR2, <3.9 mmol/L), and (f) time below range 2 (TBR2, <3.0 mmol/L).

Mean ranks across activity groups show a difference in distribution for HbA1c (*p* < 0.001), TIR (*p* < 0.001), TAR (*p* < 0.001), TAR2 (*p* < 0.001). The TBR and TABR2 mean ranks showed equal distribution across activity groups (Table 2).  Figure 2 demonstrates that the moderate activity group had a more favourable HbA1c (52.0 vs. 59.4 mmol/mol, *p* < 0.001), TIR (68.0% vs. 51.1%, *p* < 0.001), TAR (29.9% vs. 45.3%, *p* < 0.001), and TAR2 (7.6% vs. 16.1%, *p* < 0.001) when compared to the low activity group. The moderate activity group had a more favourable TIR (68.0% vs. 59.7%, *p* < 0.001), TAR (29.9% vs. 38.3%, *p* < 0.001), and TAR2 (7.6% vs. 11.0%, *p* < 0.001) compared to the mild activity group. The mild activity group had improved HbA1c (54.2 vs. 59.4 mmol/mol, *p* < 0.001) TIR (59.7% vs. 51.1%, *p* < 0.001), TAR (38.3% vs. 45.3%, *p* < 0.01), and TAR2 (11.0% vs. 16.1%, *p* < 0.05) compared to the low activity group.

## 5. Discussion

The results demonstrate that moderate-intensity activity can be incorporated into CGM-structured education programmes to aid improved glycaemic control without increasing the risk of hypoglycaemia. Most importantly, our findings suggest that the use of moderate-intensity activity to lower hyperglycaemia between meals is feasible in a real-world setting. Ideally, a cross-over randomised clinical trial of activity vs. insulin for correction of hyperglycaemia would be required to make robust recommendations. Nonetheless, given the lack of such studies, the current ISPAD 2022 guidance does support the use of exercise to lower hyperglycaemia (Grade B evidence) [5]. Our report adds to the limited literature and clarifies the feasibility of incorporating such strategies into structured education programmes.

The higher TIR achieved by participants using increasing durations of moderate-intensity activity between meals to lower hyperglycaemia using 3 months of CGM data reported here corroborates with the previous association based on 2 weeks of CGM data [14]. The moderate activity group achieved the ISPAD 2022 HbA1c target of less than 53 mmol/mol [18] and were very close to achieving the international consensus target of 70% TIR [19]. The significant improvement in TIR when increasing from low-to-mild and mild-to-moderate activity suggests a dose–response relationship to lower hyperglycaemia. The use of moderate-intensity activity to lower hyperglycaemia between meals is favoured by males and used less by CYPD from the most deprived socio-economic groups and from younger age groups. The bias towards fewer females using activity to lower hyperglycaemia may be multifactorial, including a lack of support from peers and family [20]. Female CYPD may be encouraged less to use activity to lower high blood glucose, following societal norms of expecting females to undertake less activity [20]. Higher socio-economic status is associated with more leisure time activity [21], which may explain the socio-economic bias between the groups. Anecdotally, many of the CYPD in the Moderate group reported undertaking an activity with a family member. Further qualitative work is required to better understand the barriers to use for these groups to enable targeted interventions.

The improvements in TIR did not come at the cost of increasing TBR or TBR2 in our cohort given the cautious use of the minimum effective dose of activity while taking into consideration the trend arrows. The teaching is predicated on aiming for just below 10.0 mmol/L with 10–15 min activity dropping the glucose level by ∼2.0 mmol/L considering the trend arrows (GAME, Figure 1). In contrast, Riddell et al. [1] reported hypoglycaemia in 17% of participants undertaking 45–60 min of moderate-intensity activity with a starting glucose level above 10.6 mmol/L. The differing outcomes are most likely due to the durations of activity undertaken. Our results demonstrate that prescribing shorter durations of activity and taking into account the trend arrows to personalise, minimises the risk of hypoglycaemia. Moreover, shorter durations may present less of a barrier to regular implementation in day-to-day life. Additionally, the lag time associated with CGM readings in the presence of prandial insulin and exercise must be acknowledged when educating on the risks of hypoglycaemia. One study found the CGM readings to be on average 1.1 mmol/L higher than blood glucose when blood glucose is <3.9 mmol/L, with the lag time increasing from 5 to ∼12 min, after 1 hr of activity in the presence of prandial insulin [22]. The lag reinforces the importance of only aiming to lower just below 10.0 mmol/L to prevent missing hypoglycaemia due to prolonged lag time. However, improving time in a tight range (3.9–7.8 mmol/L) without causing hypoglycaemia might be possible by aiming for less than 8.0 mmol/L. We are currently teaching to aim for less than 8.0 mmol/L in our HCL education programme.

The associations between increasing the use of moderate-intensity activity to lower hyperglycaemia between meals and improvements in HbA1c, TAR, and TAR2 further supports its efficacy in improving overall glucose control. The between-groups analysis supports the notion that a stepwise approach to incremental usage of activity will reap glycaemic benefits. Expediting insulin absorption from the sub-cutaneous tissue [23], increasing blood flow and insulin delivery to the muscles [24], and slowing hepatic and renal degradation of insulin through reduced blood flow [25] explains the rapid glucose-lowering effect of activity in the presence of bolus insulin. Also, activity induces muscle GLUT-4 translocation independent of insulin that increases glucose uptake in the absence of prandial insulin [26].

Education must include a discussion regarding the variability in glucose response to activity and other important safety and practical implementation aspects. There is significant variability in the glucose response to exercise after meals between individuals [1]. The variability between individuals is caused by numerous factors, including age, gender, and fitness level [27] and also the C-peptide status [28]; hence CYPD diagnosed within 2 years were excluded in our cohort. Some of the key factors causing intra-individual variability are the amount of insulin on board [29–31], time of day [32], and menstrual cycle status [27]. The variability in glucose response in individuals with T1D is well established. Hence, achieving effective glycaemic control using activity requires the individual to gain experience through trial and error. From a safety perspective, if the glucose level is above 14.0 mmol/L the CYPD must be educated to test for blood ketones and take appropriate action using the ISPAD 2022 exercise guidelines [5]. Appropriateness of the use of activity must be negotiated with the CYPD, for instance to avoid discrimination during school hours. Furthermore, we strongly encourage a family approach, on the understanding that activity after eating is beneficial to post-meal glucose levels in people without diabetes [11]. Family activities such as walking the dog, dancing to YouTube, a game of tag, and gardening are promoted.

The main limitation of the study is that the activity duration was self-reported in retrospect, which was not verified by activity monitors. Nonetheless, this report provides an honest account from users on the real-world utility of activity in lowering blood glucose between meals. Future trials should incorporate activity monitors to accurately record the duration and intensity of activity. We assessed the use of activity to lower hyperglycaemia 6 months after education when motivation levels are high. It is possible that usage may decrease overtime. We, therefore, use the GAME infographic to reinforce the message at each clinic visit. In future studies, it will be useful to explore if factors such as BMI, prior sports participation, day of the week, and home vs. school environment influence the uptake of activity to lower hyperglycaemia.

## 6. Conclusion

Increasing the use of moderate-intensity activity to lower hyperglycaemia between meals is associated with improved glucose control without increasing the risk of hypoglycaemia for CYPD. Teaching the incorporation of short bursts of moderate-intensity activity to lower hyperglycaemia between meals into diabetes education programmes is feasible. While the strategies may not be adopted by all, in those who do there is improved glycaemia.

## Figures and Tables

**Table 1:** Baseline study population demographics.

Characteristic	Low activity group mean (SD) or *n* (%)	Mild activity group mean (SD) or *n* (%)	Moderate activity group mean (SD) or *n* (%)	*p* Value
Participants	37 (30%)	43 (34%)	45 (36%)	
Age (years)	10.85 (4.15)	13.61 (3.26)	11.07 (2.28)	**0.003**
Gender				
Male	11 (30%)	23 (54%)	19 (42%)	**0.035**
Female	26 (70%)	20 (46%)	26 (58%)	
Duration of diabetes (years)	6.79 (3.67)	7.07 (3.88)	7.04 (3.52)	
Baseline HbA1c (mmol/mol)	60.00 (7.01)	59.29 (11.07)	56.68 (6.88)	0.091
Therapy type				
Insulin pump	25 (68%)	22 (51%)	28 (62%)	0.209
Multiple daily injections	12 (32%)	21 (49%)	17 (38%)	
Ethnicity				
White	17 (46%)	20 (47%)	20 (44%)	0.683
Asian	13 (35%)	13 (30%)	14 (31%)	
Black	7 (19%)	4 (9%)	4 (9%)	
Other	0 (0%)	6 (14%)	7 (16%)	
Socio-economic status				**<0.001**
Most deprived	24 (64%)	12 (28%)	13 (29%)	
Second most deprived	7 (19%)	11 (26%)	9 (20%)	
Third most deprived	4 (11%)	12 (28%)	16 (36%)	
Second least deprived	2 (6%)	3 (7%)	5 (11%)	
Least deprived	0 (0%)	5 (11%)	2 (4%)	

Bold values signify statistically significant.

**Table 2:** Outcome measures by activity groups.

Outcome measure	Category	*n*	Mean (SD)	Mean rank	*p* Value
HbA1c (mmol/mol)	Low	37	59.42 (7.01)	85.64	
	Mild	43	54.26 (6.85)	59.58	
	Moderate	45	51.98 (5.90)	47.66	
	Total	**125**	54.86 (7.20)		**<0.001**
Time in range	Low	37	51.11 (8.74)	35.45	
(TIR, 3.9–10.0 mmol/L)	Mild	43	59.71 (10.66)	60.78	
Percentage	Moderate	45	67.91 (10.28)	87.78	
	Total	**125**	60.00 (12.09)		**<0.001**
Time above range	Low	37	45.30 (10.65)	87.43	
(TAR, >10.0 mmol/L)	Mild	43	38.34 (10.55)	65.79	
Percentage	Moderate	45	29.93 (10.85)	40.24	
	Total	**125**	37.38 (12.36)		**<0.001**
Time above range 2	Low	37	16.14 (9.81)	84.28	
(TAR, >13.9)	Mild	43	11.03 (6.09)	66.77	
Percentage	Moderate	45	7.56 (4.71)	41.90	
	Total	**125**	11.19 (7.71)		**<0.001**
Time below range	Low	37	2.58 (1.71)	69.65	
(TBR, <3.9 mmol/L)	Mild	43	2.32 (1.94)	59.65	
Percentage	Moderate	45	2.16 (1.53)	60.73	
	Total	**125**	2.30 (1.72)		0.408
Time below range 2	Low	37	0.57 (0.48)	71.00	
(TBR2, <3.0 mmol/L)	Mild	43	0.56 (0.76)	60.63	
Percentage	Moderate	45	0.38 (0.32)	58.69	
	Total	**125**	0.47 (0.56)		0.265

Bold values signify statistically significant.

## Data Availability

John Pemberton sits on the Advisory Board for ROCHE.

## References

[1] Riddell M. C., Zaharieva D. P., Tansey M. (2019). Individual glucose responses to prolonged moderate intensity aerobic exercise in adolescents with type 1 diabetes: the higher they start, the harder they fall. *Pediatr Diabetes*.

[2] McMahon S. K., Ferreira L. D., Ratnam N. (2007). Glucose requirements to maintain euglycemia after moderate-intensity afternoon exercise in adolescents with type 1 diabetes are increased in a biphasic manner. *The Journal of Clinical Endocrinology & Metabolism*.

[3] Jabbour G., Henderson M., Mathieu M.-E. (2016). Barriers to active lifestyles in children with type 1 diabetes. *Canadian Journal of Diabetes*.

[4] Parent C., Lespagnol E., Berthoin S. (2023). Barriers to physical activity in children and adults living with type 1 diabetes: a complex link with real-life glycemic excursions. *Canadian Journal of Diabetes*.

[5] Adolfsson P., Taplin C. E., Zaharieva D. P. (2022). ISPAD clinical practice consensus guidelines 2022: exercise in children and adolescents with diabetes. *Pediatr Diabetes*.

[6] Johnson B., Norman P., Sanders T. (2019). Working with Insulin, carbohydrates, ketones and exercise to manage diabetes (WICKED): evaluation of a self-management course for young people with type 1 diabetes. *Diabetic Medicine*.

[7] Price K. J., Knowles J. A., Fox M. (2016). Effectiveness of the kids in control of food (KICk–OFF) structured education course for 11–16 year olds with type 1 diabetes. *Diabetic Medicine*.

[8] D’Souza R. S., Ryan M., Hawkes E. (2021). Questionnaire-based service evaluation of the efficacy and usefulness of SEREN: a structured education programme for children and young people diagnosed with type 1 diabetes mellitus. *BMJ Open Quality*.

[9] Hajat C., Hasan A., Subel S., Noach A. (2019). The impact of short-term incentives on physical activity in a UK behavioural incentives programme. *npj Digital Medicine*.

[10] Barte J. C. M., Wanda Wendel-Vos G. C. (2017). A systematic review of financial incentives for physical activity: the effects on physical activity and related outcomes. *Behavioral Medicine*.

[11] Manohar C., Levine J. A., Nandy D. K. (2012). The effect of walking on postprandial glycemic excursion in patients with type 1 diabetes and healthy people. *Diabetes Care*.

[12] Tansey M. J., Tsalikian E., Beck R. W. (2006). The effects of aerobic exercise on glucose and counterregulatory hormone concentrations in children with type 1 diabetes. *Diabetes Care*.

[13] Pemberton J. S., Kershaw M., Dias R. (2021). DYNAMIC: Dynamic glucose management strategies delivered through a structured education program improves time in range in a socioeconomically deprived cohort of children and young people with type 1 diabetes with a history of hypoglycemia. *Pediatr Diabetes*.

[14] Pemberton J. S., Barrett T. G., Dias R. P., Kershaw M., Krone R., Uday S. (2022). An effective and cost-saving structured education program teaching dynamic glucose management strategies to a socioeconomically deprived cohort with type 1 diabetes in a VIRTUAL setting. *Pediatr Diabetes*.

[15] NICE (National Institute for Health and Care Excellence) (August 2015). Diabetes (type 1 and type 2) in children and young people: Diagnosis and management. https://www.nice.org.uk/guidance/NG18.

[16] (2019). English Indices of Deprivation 2019. https://www.gov.uk/government/publications/english-indices-of-deprivation-2019-technical-report.

[17] Birmingham and Solihull Clinical Care Commissioning Group (2019). Clinical commissioning policy for continuous glucose monitoring in diabetes. https://www.birminghamandsolihullccg.nhs.uk/about-us/publications/policies/2490-policy-for-continuous-glucose-monitoring-in-diabetes/file.

[18] de Bock M., Codner E., Craig M. E. (2022). ISPAD clinical practice consensus guidelines 2022: glycemic targets and glucose monitoring for children, adolescents, and young people with diabetes. *Pediatr Diabetes*.

[19] Battelino T., Danne T., Bergenstal R. M. (2019). Clinical targets for continuous glucose monitoring data interpretation: recommendations from the international consensus on time in range. *Diabetes Care*.

[20] Duffey K., Barbosa A., Whiting S. (2021). Barriers and facilitators of physical activity participation in adolescent girls: a systematic review of systematic reviews. *Frontiers in Public Health*.

[21] Stalsberg R., Pedersen A. V. (2018). Are differences in physical activity across socioeconomic groups associated with choice of physical activity variables to report?. *International Journal of Environmental Research and Public Health*.

[22] Zaharieva D. P., Turksoy K., McGaugh S. M. (2019). Lag time remains with newer real-time continuous glucose monitoring technology during aerobic exercise in adults living with type 1 diabetes. *Diabetes Technology & Therapeutics*.

[23] Pitt J. P., McCarthy O. M., Hoeg-Jensen T., Wellman B. M., Bracken R. M. (2020). Factors influencing insulin absorption around exercise in type 1 diabetes. *Frontiers in Endocrinology*.

[24] Joyner M. J., Casey D. P. (2015). Regulation of increased blood flow (hyperemia) to muscles during exercise: a hierarchy of competing physiological needs. *Physiological Reviews*.

[25] Duckworth W. C., Bennett R. G., Hamel F. G. (1998). Insulin degradation: progress and potential. *Endocrine Reviews*.

[26] Sylow L., Kleinert M., Richter E. A., Jensen T. E. (2017). Exercise-stimulated glucose uptake – regulation and implications for glycaemic control. *Nature Reviews Endocrinology*.

[27] Yardley J. E., Brockman N. K., Bracken R. M. (2018). Could age, sex and physical fitness affect blood glucose responses to exercise in type 1 diabetes?. *Frontiers in Endocrinology*.

[28] Taylor G. S., Smith K., Capper T. E. (2020). Postexercise glycemic control in type 1 diabetes is associated with residual *β*-cell function. *Diabetes Care*.

[29] Iscoe K. E., Riddell M. C. (2011). Continuous moderate-intensity exercise with or without intermittent high-intensity work: effects on acute and late glycaemia in athletes with type 1 diabetes mellitus. *Diabetic Medicine*.

[30] Moser O., Tschakert G., Mueller A. (2015). Effects of high-intensity interval exercise versus moderate continuous exercise on glucose homeostasis and hormone response in patients with type 1 diabetes mellitus using novel ultra-long-acting insulin. *PLOS ONE*.

[31] Rabasa-Lhoret R., Bourque J., Ducros F., Chiasson J.-L. (2001). Guidelines for premeal insulin dose reduction for postprandial exercise of different intensities and durations in type 1 diabetic subjects treated intensively with a basal-bolus insulin regimen (ultralente-lispro). *Diabetes Care [Internet]*.

[32] Yardley J. E. (2022). Reassessing the evidence: prandial state dictates glycaemic responses to exercise in individuals with type 1 diabetes to a greater extent than intensity. *Diabetologia*.

